# Characterising Uncertainty in Expert Assessments: Encoding Heavily Skewed Judgements

**DOI:** 10.1371/journal.pone.0141697

**Published:** 2015-10-30

**Authors:** Rebecca A. O’Leary, Samantha Low-Choy, Rebecca Fisher, Kerrie Mengersen, M. Julian Caley

**Affiliations:** 1 Australian Institute of Marine Science, The UWA Oceans Institute (M096), 35 Stirling Highway, Crawley, Western Australia 6009, Australia; 2 School of Mathematical Sciences, Science and Engineering Faculty, Queensland University of Technology, GPO Box 2434, Brisbane, Queensland 4001, Australia; 3 Australian Institute of Marine Science, PMB 3 Townsville MC, Townsville, Queensland 4810, Australia; New York University School of Medicine, UNITED STATES

## Abstract

When limited or no observed data are available, it is often useful to obtain expert knowledge about parameters of interest, including point estimates and the uncertainty around these values. However, it is vital to elicit this information appropriately in order to obtain valid estimates. This is particularly important when the experts’ uncertainty about these estimates is strongly skewed, for instance when their best estimate is the same as the lowest value they consider possible. Also this is important when interest is in the aggregation of elicited values. In this paper, we compare alternative distributions for describing such estimates. The distributions considered include the lognormal, mirror lognormal, Normal and scaled Beta. The case study presented here involves estimation of the number of species in coral reefs, which requires eliciting counts within broader taxonomic groups, with highly skewed uncertainty estimates. This paper shows substantial gain in using the scaled Beta distribution, compared with Normal or lognormal distributions. We demonstrate that, for this case study on counting species, applying the novel encoding methodology developed in this paper can facilitate the acquisition of more rigorous estimates of (hierarchical) count data and credible bounds. The approach can also be applied to the more general case of enumerating a sampling frame via elicitation.

## Introduction

This paper addresses the problem of eliciting from an expert both point and interval estimates of a parameter of interest, where the expert data is heavily skewed. The use of information elicited from experts to complement and inform statistical analyses is growing in popularity, particularly with the development of rigorous methods for obtaining such information. Elicited estimates of variables of interest may be useful, for example, when data gaps exist, such as in the *post-Normal science* situation in which inference is required before the data are available [1] or if the sampling effort required to collect the required data is impractical or too expensive [2]. Whilst structured approaches for quantifying such expert knowledge have become more readily available in ecology [3–5], these generally encode expert knowledge and their uncertainty using symmetric or moderately skewed forms of distributions. There is little literature on elicitation of highly skewed distributions.

This problem of encoding skewness arises in the general context of eliciting hierarchically structured or nested parameter estimates and can be exacerbated if there is interest not only in the individual parameters but also in the aggregation of the hierarchical information. Consider the simple case of estimation of a total value, *X*, based on *X*_0_ the known value and *X*_*j*_, *j* = 1, ‥, *J* estimates of additional components, such that

$$\begin{array}{c} X=X_{0}+\sum_{j=1}^{J}X_{j}. \end{array}$$ (1)

The particular problem that motivated this study was the estimation of coral reef biodiversity. This paper describes, and examines, in more detail the methodology used by Fisher *et al*. [6] to estimate the global number of species on coral reefs, using the methodology presented in this paper, including estimates of the number of named species, discovered but unnamed species and undiscovered species. In addition estimates number of species for 39 higher-level taxa. These estimates were obtained from experts (taxonomists) during one at a time face-to-face interviews using Fisher *et al*. [7] elicitation software.

The total number of species can be estimated in each of multiple taxonomic classifications and then summed across taxa [8–10]. Thus an estimate of the number of species that reside on coral reefs, *X*, can be accumulated over taxonomic groups *j*, as described in Eq (1). Within taxonomic group *j*, species richness may be further disaggregated:

$$\begin{array}{c} X_{j}=X_{0j}+\sum_{m}X_{jm}. \end{array}$$ (2)

In the marine biodiversity context, databases such as WoRMS [11] can typically provide an empirical estimate, providing a natural offset *X*_0*j*_. However due to the so-called ‘taxonomic impediment’ [12], it is well-known that *X*_0*j*_ may substantially underestimate the true value *X*_*j*_ due to the number of species yet to be catalogued and yet to be discovered. By definition no data are available on these components, so they provide obvious candidates for elicitation from experts. Hence richness in a taxonomic group *j* may usefully be conceptualized as being partitioned into three non-overlapping components [7]:

$$\begin{array}{c} X_{j}=K_{j}+D_{j}+U_{j}, \end{array}$$ (3)

where *K*_*j*_ represents the *known* total number of described and named species in the *j*th taxon of interest, *D*_*j*_ accounts for species that have been *discovered* but are as yet unnamed, and *U*_*j*_ identifies the number of species that are *undiscovered* and therefore unnamed. This is consistent with recommendations e.g. [13] that elicitation to quantify a complex entity (such as the number of species on coral reefs) will benefit from decomposition into components that an expert can more readily comprehend, and thus more accurately estimate.

By expansion, components *K*, *D* and *U* may themselves need to be disaggregated, to better reflect the ‘kernels’ of expert knowledge about species richness:

$$\begin{array}{c} \;K_{j}=\sum_{k}K_{jk},\;D_{j}=\sum_{i}D_{ji},\;U=\sum_{\ell }U_{j\ell }. \end{array}$$ (4)

The known component may need to be disaggregated into sub-components *K*_*jk*_ across several categories, which may reflect separate database records for sub-species or research studies. The discovered component can be disaggregated into *D*_*ji*_, *i* = 1, 2, 3 representing (1) cryptic, (2) genetically distinct, and (3) morpho-species in that taxon. The unknown sub-components *U*_*j*ℓ_, *i* = 1, 2, 3 may arise due to: (1) sampling inefficiency, when species that have not been collected in areas previously sampled; or species that remain undiscovered because (2) particular locations or (3) habitats that remain relatively or completely unsampled (see [7] for further details).

In addition to point estimates of *D*_*ji*_ and *U*_*j*ℓ_, it is also important to capture interval estimates, reflecting the expert assessment of the plausible range of values for each component. Skewness in the interval assessments may be induced by the hierarchical structure of the counts. Some researchers are considered ‘splitters’ and tend to over-differentiate species, which at some later date may be resolved via aggregation, hence reducing the apparent richness. Thus the current reported count may provide an upper bound on the number of species in the taxon, leading to elicitation of *positive* skewness in the plausible range of species richness. Conversely, other researchers are considered ‘lumpers’ and may under-differentiate species, which will later lead to an apparent increase in richness. So current reported counts may provide a lower bound on the number of species in the taxon, leading to *negative* skewness in the plausible range of richness. *Negative* skewness could also arise, for example when individual species within a large taxon such as Family or Order have been assigned more than one name by different taxonomists. More *extreme* skewness (positive or negative) could also arise if the best estimate given by a taxonomist coincides with the known published number [11].

There are many analogues of this type of problem. For example, estimation of the population size of a country involves estimation of subgroups, some of which are more difficult to enumerate using standard census techniques, such as the number of homeless people e.g. [14, 15] or minority people in remote areas [16]. Although some methods exist for obtaining ballpark values for these subgroups e.g. [17], experts could also provide informative estimates of the required enumerations. With continued divisions of the subgroups, the associated expressions of uncertainty would be expected in at least some situations to become more skewed.

This setup also extends more generally to that of estimating the size of a sampling frame in which the population is hierarchically stratified. The above decomposition into components, which are estimated with different levels of uncertainty, provides an obvious mechanism for compiling an overall estimate of the size of the sampling frame. As indicated above, the hierarchical structure may impose constraints on the aggregation of components. For instance, in a landscape ecology context, ecoregions are hierarchically defined, and since these form a non-overlapping partition of the space, the number of regional ecosystem units within an ecoregion must ‘add up’. In the species richness case study, this hierarchy is an inherent feature of taxonomic classes of classification, since all species belong to a series of higher taxonomic groups, from genus to kingdom.

Positive skewness in elicited values has been captured using the lognormal e.g. [18, 19], Poisson [20], negative binomial [21] and Beta e.g. [22, 23] distributions, as well as the quantile-defined Davies distributions [24]. Although they can be easily generalised to other data types, the above problems involve the elicitation and aggregation of point and interval estimates of counts, so a natural choice might be a distribution such as the Poisson, Negative Binomial or Multinomial. Single parameter distributions such as the Poisson make it difficult to separate the expert’s best estimate from their uncertainty. Indeed, in our experience there are difficulties in using any of these distributions for elicitation of highly skewed intervals because of the non-independence of the mean and variance [25]. Thus in this paper we consider the more flexible lognormal and mirror lognormal distributions to describe both left and right asymmetry, or alternatively the normal distribution for (reasonably) symmetric estimates. Despite the relationship between the mean and variance, the Beta distribution provides a very flexible shape that is capable of capturing high skewness. For this reason, we also compare the set of distributions based on the normal with the scaled Beta distribution. To our knowledge, the mirror lognormal distribution has not previously been used in the context of elicitation. We explore their potential to encode highly skewed expressions of the plausible range of values expressed by experts.

Eliciting expert opinion and encoding these values using a Beta distribution is a common approach; see, for example, the literature reviews in [22] and [23]. There are various software packages and routines that perform this encoding. We discuss here three examples, the R prevalence package, Elicitator [26] and the MATCH Uncertainty Elicitation Tool [27].

The functions betaExpert and betaPERT within the R prevalence package can fit 2— and 4—parameter Beta distributions respectively. The expert provides a minimum value, most likely estimate (mode) or mean and maximum value. For the betaExpert function the lower and upper numbers must lie between 0 and 1, whereas for betaPERT the range can be anywhere on the real line. The betaExpert function uses least squares to estimate *α* and *β*. When the best guess is set to zero or one (and thus the expert only provides either an upper or lower estimate), then the corresponding *α* or *β* is set to one and least squares is used to find the unknown parameter. For the 4-parameter Beta distribution (betaPERT function) the *α* and *β* are calculated using the simple estimates proposed by [28]; see also [29]. Both of these functions allow the elicited best guess to be equal to the minimum or maximum value.

Morris *et al*. [27] developed a web-based elicitation tool called the MATCH Uncertainty Elicitation Tool. This tool provides five elicitation methods to estimate the expert’s probability distribution for a variable *X*, namely the roulette method (based on the proportion of chips placed in bins spanning the range of *X*), the quartile method (based on eliciting the median, lower quartile and upper quartile of *X*), the tertile method (based on eliciting the tertiles of *X*), the probability method (based on eliciting three probabilities such as P(0 < *X* < 0.25), P(0.75 < *X* < 1), P(0 < *X* < 0.5) if *X* is between 0 and 1) and the hybrid method (based on eliciting the median and two probabilities). Six distributions are available for describing the elicited values, namely the normal, *t*, beta (scaled if the limits are not 0 and 1) gamma, log normal and log *t*. The software reports which distribution best fits the judgement; highly skewed judgements can lead to choice of the (scaled) Beta distribution. In the methodology underlying the MATCH tool, it was assumed that the elicited median would not equal either the elicited minimum or maximum, and hence does not allow for extreme skewness.

James *et al* [26] developed an elicitation software called Elicitator, which quantifies expert knowledge and incorporates this into a prior model in Bayesian regression. This tool can be applied to the problem of asking the expert about the probability of presence at a number of sites/observations with known covariate values (such as habitat and environmental variables). For each covariate, the expert provides the lower and upper bounds (99 or 95% credible interval), then specifies another interval such as the mode. These values are encoded into the Beta distribution using iteratively reweighted least squares.

This paper will describe a methodology for capturing highly positively or negatively skewed counts. In our previous work [7], Normal and lognormal distributions were encoded when the expert provided answers as counts or multiplicative factors, and Beta distributions were encoded to elicited percentages. The details of encoding these distributions for elicited data were not specified in Fisher *et al* [7]. In this paper, we provide these details and also present an alternative distribution, the scaled Beta, which can be encoded to all elicited numbers. In addition, we address the overall problem of aggregating elicited counts across strata while respecting the laws of conservation of counts within hierarchies. More detailed evaluation and comparison of different modelling choices is provided in this paper, which could not be examined in detail in Fisher *et al* [7]. This comparative analysis will investigate the choice of statistical distributions for the type of extreme cases encountered in the case study data. The methods are applied to the reef biodiversity case study, in which we are estimating the total number of species over a broad range of taxonomic groups.

## Methods

### Elicitation protocol

The choice of summary statistics used to describe the distribution and the order in which these statistics are elicited are both important considerations [3, 30, 31]. Following the recommendations of these authors, we adopted an ‘outside-in’ protocol for elicitation, which starts by eliciting a plausible interval of values, and ends by eliciting a point estimate. Under our protocol, the following information was sequentially obtained: (i) extrema, typically as absolute lower and upper limits, (ii) quantiles at fixed cumulative probabilities, as realistic lower and upper limits, and (iii) the mode, as the most plausible value. So for an initial estimate, the summary statistics elicited were, in order [7]: (i) minimum (*M*_*L*_) and maximum (*M*_*U*_), so that the expert is 100% sure that the number is within this range; (ii) more realistic lower (*L*) and upper (*U*) bounds, and the uncertainty/sureness (*P*) around these bounds, elicited by asking how sure they are that the real number lies within these bounds; and (iii) their best estimate (*B*), the number they thought was most plausible. Following [24], *B* was encoded as a mode, since this value was elicited using a phrase such as, “what is the most likely value?”

A further statistical decision is the unit of elicitation. In the case study, we allowed the expert to choose to provide answers using one of three units. For an absolute estimate, a count would indicate the number of actual additional species that would add to the previous hierarchy’s estimate of the total number of species on coral reefs, whereas a relative estimate could be provided via either a percentage (of the number of named species) or a multiplicative factor (e.g. twice as many as the named species). This was designed to allow the expert to provide information in units with which they are most comfortable [32].

### Statistical encoding of the elicited information

When encoding the elicited values into statistical distributions, it is useful to be able to capture both uncertainty as well as best estimates. This requires that a distribution has at least two parameters [24]. For example, in the case study, depending on the taxonomic group, regardless of the best estimate of the number of species in a taxon, the variance of the elicited number of species can range from very small (the total number of species is known and there is little uncertainty around this number) to very large (the total number of species is unknown and the uncertainty around how small/large this number could be is large).

In the elicitation software developed by [7], the Normal and lognormal distributions were fitted when the expert provided answers as counts or multiplicative factors. However, when the expert answered as a percentage, a Beta distribution was fitted to the corresponding proportion. An alternative and more flexible approach not considered by Fisher *et al*. [7] is to fit a scaled Beta distribution to all numbers elicited from the expert. This provides a common platform for encoding the full range of cases, from symmetric to extreme left and right skewed elicited values, and has the added advantage of being applicable across a wide range of units of elicitation including the three used in the case study. The upper number is a suitably large real value in practice, where the expert considers that there is negligible chance that it will be exceeded. While Normal/lognormal distributions can be fitted to percentages, these distributions need to be truncated so that the numbers range between zero and 100 percent, or else the percentages would have to be transformed, for example via a log odds transformation. Below is a description of how the elicited numbers are fitted to Normal, lognormal, Beta and scaled Beta distributions.

#### Skewness

We consider statistical distributions with two parameters to encode expert assessments comprising three summary statistics (*B*, *L*, *U*) for a specified cumulative probability *P*. A distribution with a single parameter would not be flexible enough to capture spread, reflecting expert uncertainty, as well as location, representing the best estimate. In contrast, in most cases a distribution with three parameters would fit the limited elicited data too closely, leaving no room for elicitation error. Obvious exceptions to this include the case in which an expert provides a symmetric interval about the mode, in which case three elicited judgements can be fitted perfectly with a two-parameter distribution. This leaves several choices for two-parameter distributions, which can all be constructed to have the same location and similar spread. Skewness provides a useful way to distinguish the differences among these. Since quantiles are pivotal to fitting the expert uncertainty, we consider the quantile-based definition of skewness [33], which reflects symmetry of the encoded quantiles around the encoded median:

$$\begin{array}{c} \gamma_{B}\left(p\right)=\frac{q(p)-q(0.50)+q(1-p)-q(0.50)}{q(p)-q(1-p)}. \end{array}$$ (5)

This intuitive measure of the relative distance of each quantile from the *p*th quantile was documented at least a century ago by [34] (hence the *B* subscript). Unlike the moment-based measure of skewness, the quantile based measure is bounded by −1 and +1.

#### Encoding via least squares

Encoding a two-parameter distribution such as the Normal or Beta is typically addressed via estimating equations to provide a *deterministic* encoding of 2 parameters from the same amount of data [23]. The least squares fitting approach has been utilized previously by these authors [24–26, 35] and those of MATCH [27]. To allow for some uncertainty in elicitation, we may slightly expand the estimation problem to encoding two parameters from three pieces of data, hence defining a statistical rather than deterministic approach. Let *θ* denote a *K*-dimensional vector of parameters associated with the probability distribution used to encode the expert estimates. For a given distribution, the aim is to find the parameter vector *θ* that best fits the elicited quantities. Denote the elicited ‘data’ by *D* with encoded quantities *D*(*θ*) corresponding to their expected values, so that for *K* = 3:

$$\begin{array}{c} D=\left[B,L,U\right]\;\text{and}\;E\left[D\right]=D\left(\theta \right)=[\mu \left(\theta \right),Q\left(p_{L};\theta \right),Q\left(p_{U};\theta \right)], \end{array}$$ (6)

where *Q*(*p*; *θ*) = *F* ^−1^(*p*; *θ*) is the quantile function (inverse cumulative probability distribution) evaluated at cumulative probability *p*, with parameter vector *θ*. As in [24], we assume that the elicited quantities (for a particular expert) are far enough apart that the difference between elicited and encoded values has effectively constant variance *ϕ* ^2^ after appropriate transformation. This effectively defines a regression [35]:

$$\begin{array}{c} D∼N(D\left(\theta \right),\phi^{2}). \end{array}$$ (7)

which implicitly involves *ϕ* as a parameter for uncertainty in elicitation. A least squares approach minimizes the distance *ρ* between the elicited and encoded quantities, as measured by the mean sum of squares (MSS):

$$\begin{array}{c} \hat{\theta }=\arg\min_{\theta }\text{MSS}\;\text{where}\;\text{MSS}=\frac{1}{K}\sum_{k=1}^{K}\rho \;(D_{k},D_{k}\left(\theta \right)). \end{array}$$ (8)

Straightforwardly, we use Euclidean distance with *ρ*(*y*_1_, *y*_2_) = (*y*_1_ − *y*_2_)^2^. This is appropriate when encoding a symmetric distribution such as the Normal. When encoding skewed counts, we use a log-transformation *ρ*(*y*_1_, *y*_2_) = (log(*y*_1_) − log(*y*_2_))^2^. When encoding proportions, we use a logit-transformation *ρ*(*y*_1_, *y*_2_) = (logit(*y*_1_) − logit(*y*_2_))^2^.

Given that the parameters of the Beta distribution jointly define the mean and variance, and hence the probability intervals, and given that, according to the elicitation protocol the outer limits are elicited before the mode, it is reasonable to place equal weight on the *K* = 3 summary statistics. Other arguments could be made, of course, for placing differential weights on these statistics, and a sensitivity analysis could be undertaken to assess the impact of such decisions. This was not pursued here.

The least-squares estimator provides a fast and generic approach to estimation, available in real-time, which allows instantaneous verbal or graphical feedback [3, 26]. For the coral reef study, the elicitation of the number of species in a taxon was conducted in a single sitting, so it was important to provide instantaneous feedback to experts. To implement a fast and efficient search across parameter space, the function ‘optim’ in R [36] was used to implement the [37] method to minimise the MSS.

#### Normal/lognormal Distributions

We use the usual *μ*-*σ* parameterization of the Normal distribution:

$$\begin{array}{c} X∼\text{N}\left(\mu ,\sigma \right)\Leftrightarrow \ell \left(x;\mu ,\sigma \right)=\frac{1}{\sqrt{2\pi \sigma }\exp\{\frac{{\left(x-\mu \right)}^{2}}{2\sigma^{2}}\}}. \end{array}$$ (9)

The lognormal provides a distribution of non-negative values that are positively skewed, and can be defined in terms of the Normal distribution:

$$\begin{array}{c} \log X∼\text{N}(\mu ,\sigma ;U),\;\text{defined}\;\text{only}\;\text{where}\;X>0. \end{array}$$ (10)

The mirror lognormal distribution is negatively skewed, and is constructed as the mirror image of the lognormal distribution, with reflection around an upper bound *U*:

$$\begin{array}{c} \log L+\log U-\log X∼\text{N}(\mu ,\sigma ;L,U),\;\text{defined}\;\text{only}\;\text{where}\;U\geq X\geq L. \end{array}$$ (11)

The classical definition of mirroring is defined in terms of the cumulative distribution function (cdf). In this case, log *X* ∼ *N*(*μ*, *σ* ^2^) which has cdf Φ(⋅), means that the classical mirrored distribution has cdf 1 − Φ(⋅), which is the cdf of −log*X*. Here log(*L*) + log(*U*) − log(*X*) has close to the required mirrored distribution, apart from a negligible numerical discrepancy caused by presuming that Pr(log *X* ≥ log *U*) and Pr(log *X* ≤ log *L*) are small and are of similar magnitude so ‘cancel’.

A lognormal, normal or mirror lognormal distribution can be applied if the expert’s response is in the form of a count or multiplicative factor. In the software developed by [7], if the expert’s data is positively skewed, i.e. if the respondent’s best guess *B* falls below the lower quartile *q*(0.25), then a lognormal distribution is fit. Similarly if if the expert’s data is negatively skewed, i.e. *B* falls above the upper quartile *q*(0.75) then a mirror lognormal is fit. Otherwise, if *B* falls within the inter-quartile range then the normal distribution is fit, together with a lognormal, if *B* < *q*(0.5), or the mirror lognormal, if *B* > *q*(0.5). Then the MSS is used to select the best distribution.

**Fig 1:**
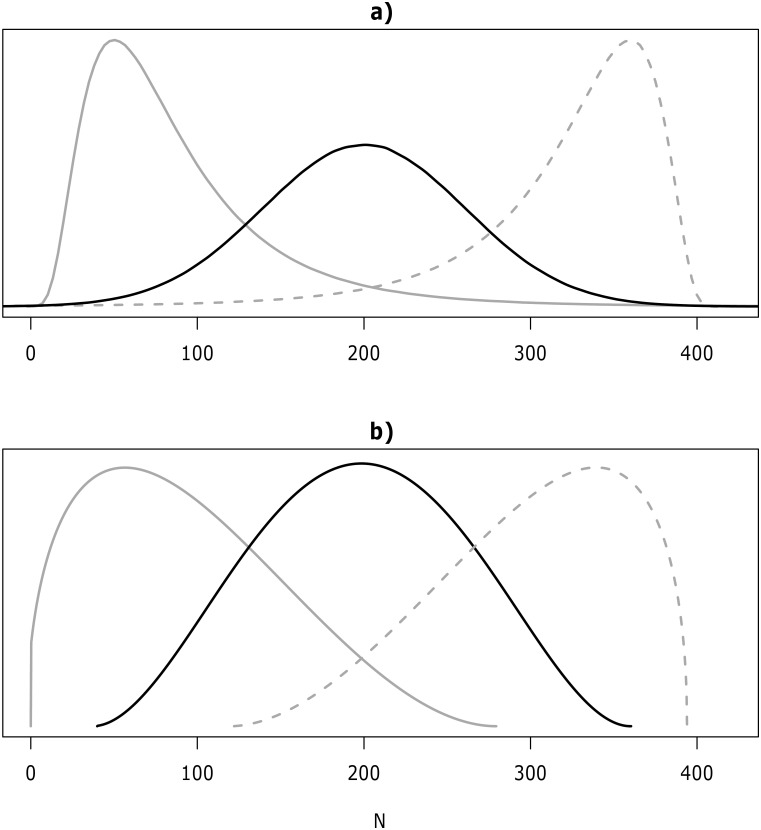
Example of three types of skewness. (a) three Normal and (b) scaled Beta distributions. Where the black line in both plots is zero skewness (*γ*_*B*_(0.5) = 0) and the elicited values are *M*_*L*_ = 80, L = 100, B = 200, U = 300, *M*_*U*_ = 320 and P = 0.9. Positive skewness is the grey line and the elicited values are *M*_*L*_ = 0, L = 10, B = 60, U = 200, *M*_*U*_ = 250 and P = 0.9; skewness for (a) lognormal is *γ*_*B*_(0.5) = 0.338 and (b) scaled Beta is *γ*_*B*_(0.5) = 0.177. Negative skewness is the grey-dashed line and the elicited values are *M*_*L*_ = 170, L = 200, B = 340, U = 380 *M*_*U*_ = 385 and P = 0.9; skewness for a) mirror lognormal is *γ*_*B*_(0.5) = −0.338 and b) scaled Beta is *γ*_*B*_(0.5) = −0.177.

For a normal distribution (Fig 1), we may encode parameters *μ*, *σ* via the least-squares approach detailed above, using the following procedure:

1. Interpret the best estimate as the mode, which in the Normal case also encodes the mean, so $\hat{\mu }=B$;
2. Use the least squares estimate of the standard deviation $\hat{\sigma }$ for fixed $\hat{\mu }=B$, using the Euclidean distance *ρ*(*y*_1_, *y*_2_) = (*y*_1_ − *y*_2_)^2^ for the MSS.

For a lognormal distribution (Fig 1), we use a similar approach

1. Interpret the best estimate as the mode $\hat{B}=e^{\mu -\sigma^{2}}$ and use this to constrain the *μ* − *σ*-space.
2. Use least squares to estimate the standard deviation, using the log-transformed Euclidean distance, with MSS_LN_ is as specified in detail in S1 Text.

Similarly least squares estimates for *μ*, *σ*, *U* can be obtained for the mirror lognormal distribution (Fig 1).

#### Beta Distribution

If the expert answers as a percentage, then a Beta distribution *Beta*(*α*, *β*) can be fitted [24]:

1. Interpret the best estimate *B* as the mode $\hat{B}=\left(\alpha -1\right)/\left(\alpha +\beta -2\right)$.
2. If the expert declares that the best estimate *B* equals the lower *L* number (i.e. extreme positive skewness), then set *β* to one and calculate *α*. Alternatively if *B* = *U* (i.e. extreme negative skewness), then set *α* to one and calculate *β*.
3. Use least squares to estimate *α* and *β*, based on the logit-transformed Euclidean distance and $MSS_{Beta}$ defined in S1 Text.

#### Scaled Beta Distribution

All elicited numbers can be fitted to a scaled Beta distribution, irrespective of the units elicited and degree of skewness, by scaling them to the range [0, 1] and fitting the Beta distribution described above. Of course, if a proportion is elicited on the full unit interval, then no scaling is required. However a proportion with minimum and maximum falling within this interval could be fit to a scaled Beta distribution. Thus the only additional step is the scaling of units elicited as numbers of multiplicative factors. Although the expert’s values of *M*_*L*_ and *M*_*U*_ could be used to effect this scaling, they can induce an unnecessarily severe reduction since they represent the 100% credible bound. The lower and upper bounds elicited can be overly conservative. For instance an expert may specify that there is negligible chance (by which they mean nearly 100% chance, i.e. at least 99% chance) that the number of species exceeds some upper bound. However, the level of conservatism is not clear until they are asked to bring their bounds in a little to reflect a more realistic upper bound (e.g. to define an upper bound exceeded with exactly 5% chance). For this reason, it is important that the lower and upper bounds are elicited, and feedback is provided, on the original scale of the number of interest.

An alternative is to calculate scaling factors Δ_*U*_ and Δ_*L*_, respectively for the lower and upper bound:

$$\begin{array}{c} \Delta_{U}=\left(\left(U-B\right)+\left(M_{U}-U\right)\right)/2\;\text{and}\;\Delta_{L}=\left(\left(B-L\right)+\left(L-M_{L}\right)\right)/2\;. \end{array}$$ (12)

Using $\tilde{M_{U}}=\Delta_{U}+U$ and $\tilde{M_{L}}=L-\Delta_{L}$, the values of *B*, *L* and *U* are converted into proportions by

$$\begin{array}{c} \begin{array}{c} \pi_{U}=1-\left(\left(\tilde{M_{U}}-U\right)/\left(\tilde{M_{U}}-\tilde{M_{L}}\right)\right)\\ \pi_{L}=1-\left(\left(\tilde{M_{U}}-L\right)/\left(\tilde{M_{U}}-\tilde{M_{L}}\right)\right)\\ \pi_{B}=1-\left(\left(\tilde{M_{U}}-B\right)/\left(\tilde{M_{U}}-\tilde{M_{L}}\right)\right). \end{array} \end{array}$$ (13)

These numbers can then be fitted to the above Beta Distribution, to encode the parameters *α* and *β*.

Fig 1 shows examples of three Normal and scaled Beta distributions. The figure shows examples of positive skewness, symmetry (*γ*_*B*_(0.5) = 0) and negative skewness. In both the positive and negative skewness examples, the scaled Beta distribution has a lower skewness value compared with the lognormal and mirror lognormal distributions.

### Aggregating values

Now we return to the main problem of obtaining an aggregate estimate of *X* in Eq 1. One approach would be to simply sum the elicited values $\hat{X}=\sum_{j}\;B_{j}$, i.e., the best guesses for each component. However, this approach does not incorporate the expert’s uncertainty, and does not account for discrepancies between elicited modes and encoded means. If all components were encoded using Normal distributions *X*_*j*_ ∼ N(*μ*_*j*_, *σ*_*j*_), then we could straightforwardly apply analytical results so that $X∼\text{N}(\sum_{j}\;\mu_{j},\sqrt{\sum_{j}\;\sigma_{j}^{2}})$. However here we potentially have finite mixtures of Normal, lognormal, mirror lognormal and Beta distributions. Thus we use simulation to estimate the expected value of *X* and the corresponding variance and desired probability intervals.

As an illustration of a simple simulation approach, in the case study and comparative study described below, 100,000 random values were drawn from each distribution. The desired aggregate number was then obtained by summing the simulated values at each iteration. The best guess and the corresponding lower and upper probability bounds were calculated from the resulting empirical distribution of summed values.

A different type of aggregation problem occurs when there are multiple experts and the goal is to estimate a single distribution which captures their combined beliefs. In such cases, often a “consensus” approach is adopted in which a panel of experts is consulted. This is a particular form of a behavioural aggregation approach [23], where group discussions lead to a consensus, similar to the moderation phase of the Delphi method e.g. [38]. The disadvantage of this consensus approach is that disagreement between experts or domination of the panel by one or more experts may lead to biased outcomes e.g. [39, 40].

A second way of combining these expert opinions is to elicit the information separately from individual experts and then combine these multiple opinions into a single distribution. This mathematical approach to aggregation [23] is particularly useful if the experts live far apart and cannot easily be assembled into an expert panel, and where, as in the case study, typically only one expert is available per component (here a taxonomic group). To estimate this consensus distribution mathematically, a common and simple method uses a mixture of experts model, via a suitably transformed weighted sum of the individual experts’ distributions. This is also known as linear or geometric pooling [23]. A third approach employs statistical modelling to consider the consensus distribution in a multi-level model [35]. In this study we found that the standard approach of linear pooling was sufficient, and flexibly could be used, either to pool distributions across experts for a particular taxon, or when summing contributions (and their uncertainty) to overall counts arising from different sources.

The most “democratic” method of assigning weights to each expert is to apply an equal weight to the opinions of each expert. Alternatively, more weight might be given to some experts over others based on their assessed level of expertise [23]. Other work [41] has developed a conceptual model that outlines criteria for defining taxonomic expertise, that could provide a basis for transparently defining weights for each expert [42]. Regardless of the weighting, the problem reduces to a sum of distributions and can be addressed using the approaches described above. For this study, it was sufficient to consider equal weighting of experts in the few cases where multiple experts had knowledge on the same taxonomic group.

### Comparative study

The comparative study was designed to evaluate the relative ability of the Normal and lognormal distributions, and the scaled Beta distribution, to fit a wide range of skewed distributions. The minimum values *M*_*L*_ were set at 0, 50, 90 and 100 and the maximum values *M*_*U*_ were 200, 210, 250 and 500. The lower and upper bounds were set at *L* = 100 and *U* = 200, respectively, with sureness values of 0.7, 0.8 and 0.9. All values of the best guess between the lower and upper limits were also tested by setting *B* to be a sequence between 100 and 200 incremented by one. The sureness, minimum and maximum values were selected based on the case study.

Estimates obtained for the sum of distributions, based on Eq 1, were also obtained for the two distributional approaches. These were compared using graphical displays of the data, fitted distributions and encoded lower, upper and mode values. The root mean square error (RMSE), calculated as the square root of the average of the squared differences between the estimated and true values, was also calculated for each distribution. This was used as a comparative measure of goodness of fit between the distributions. We also evaluated quantile-based skewness (*γ*_*B*_(0.5)) to supplement the elicited quantities by a description of the level of skewness in each scenario.

## Results

### Case study

**Fig 2:**
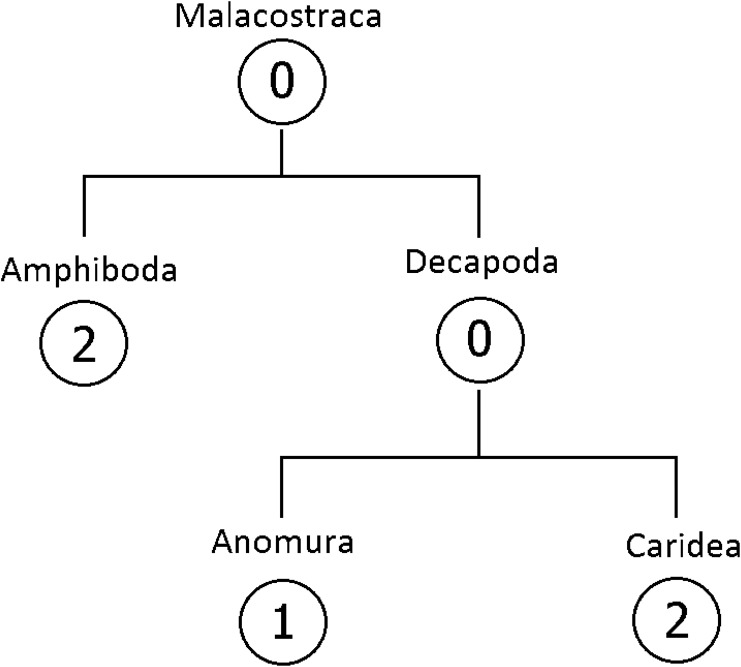
The taxonomic hierarchy of Malacostraca. The number within a circle represents the number of experts elicited from in each taxon within the class.

The case study presented in this paper involves encoding taxonomists’ estimates of species richness within the class Malacostraca, and associated uncertainty about these estimates (Fig 2). Malacostraca is the largest of six classes of crustaceans and is comprised of the orders Amphipoda and Decapoda (Fig 2). The Decapoda in turn are comprised by the Anomura and Caridea. We were able to elicit information from one expert on Anomura, and two experts for each of Caridea and Amphipoda. There was no expert available for Malacostraca and Decapoda. All elicitations took approximately 1.5 hours to complete and all taxonomists expressed confidence that the process captured well their views of the global species richness of the taxa for which we elicited their expert knowledge. Other details about the elicitation process, including feedback, are reported in [7].

For this case study, 63.3% of the elicited estimates of uncertainty were reasonably symmetric, 26.67% were positively skewed and 6.67% were negatively skewed. Of the cases with positively skewed elicited uncertainty, the majority (20% of the total) were extremely positively skewed, that is *B* = *L*. For one expert, one of the estimates was zero. From this elicited information, the aim was to calculate the total number of species in each taxon and in Malacostraca. For each taxon and expert, the elicited information for each component was fitted to both the scaled Beta and Normal/lognormal distributions.

**Fig 3:**
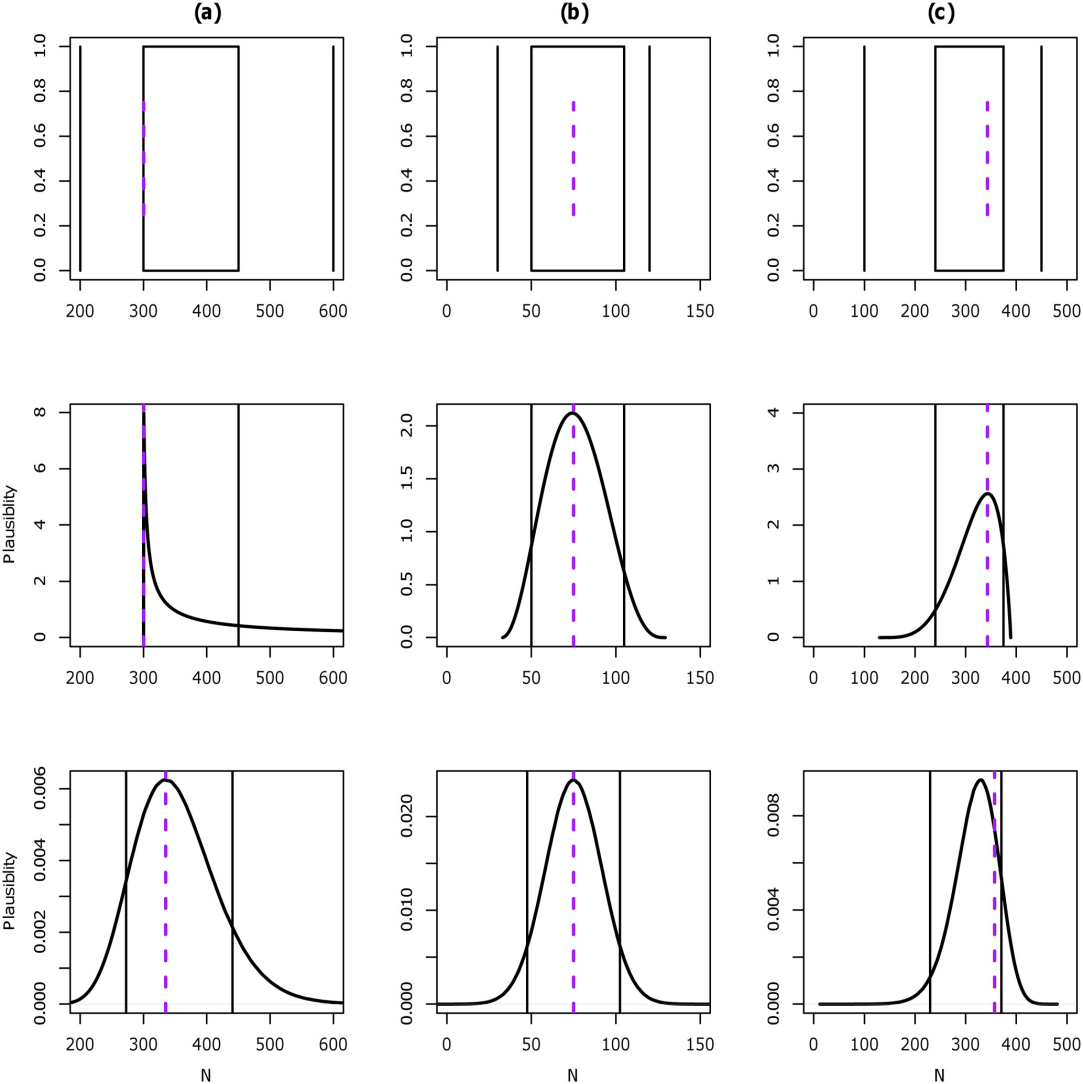
Three examples of expert data from case study (class Malacostraca) and comparison of the fits between the scaled Beta and Normal/lognormal distributions. (a) an example of extreme positively skewed expert data, where the best guess equals the lower number; (b) symmetric expert data; and (c) left skewed expert data. The first row shows box plots of the expert data; the second row shows the fitted distributions from the scaled Beta distribution, and the last row shows the fitted distributions from (a) lognormal, (b) normal and (c) mirror lognormal distributions. For the box plots (first row), the outer two lines are the minimum and maximum (expert almost 100% sure the number of species is between these two values); the box represents the lower and upper number elicited; and purple dashed line is the best guess. Second and third row shows the fitted distributions, the solid black vertical lines are the encoded lower and upper, and purple dashed line is the encoded mode.

Fig 3 shows three examples of the expert data and the performance of the scaled Beta and Normal/lognormal distributions fitted to these expert data. The first, (a), is an example of extreme positively skewed expert data (first expert for Amphipoda). In the second example, (b), the expert data are symmetric (from taxon Amphipoda). The last example, (c), illustrates negatively skewed expert data (second expert for Amphipoda). The elicited numbers (*M*_*L*_, *L*, *B*, *U*, *M*_*U*_, *P*) for these three examples were (200, 300, 300, 450, 600, 0.8), (30, 50, 75, 105, 120, 0.9) and (100, 240, 343, 375, 450, 0.9), respectively (S1 Table).

In all three examples the scaled Beta distribution provided a better fit (smaller encoding error) to the expert data compared with the Normal/lognormal distributions. For example (a), the scaled Beta distribution accurately fitted the expert’s values of *L*, *B* and *U*. However, for the lognormal, the three fitted values were skewed further right compared to the elicited values (*L* = 272.6, *B* = 334.7, *U* = 440.7). The scaled Beta distribution had a smaller positive skewness value (*γ*_*B*_(0.5) = 0.303), compared to the lognormal (*γ*_*B*_(0.5) = 0.378). The RMSE also demonstrated that the scaled Beta distribution performed better, with a value of almost zero for the Beta and 26.060 for the lognormal. For example (b), where the expert responses indicated symmetry, both the Beta and Normal distribution accurately estimated the expert’s lower, mode and upper values. The skewness measurement indicates that both distributions were close to symmetric, with scaled Beta distribution *γ*_*B*_(0.5) = 0.044 and Normal *γ*_*B*_(0.5) = 0.091. The RMSE for both distributions were small, with Beta RMSE = 7.3 × 10^−5^ and the normal RMSE = 2.041. For example (c), the Beta distribution correctly reproduced the expert data, but the mirror lognormal incorrectly encoded all three numbers, with smaller fitted values of *L* and *U* (229.9 and 370.6 respectively) and a larger fitted mode. Both distributions had similar negative skewness values at *γ*_*B*_(0.5) = −0.248 for scaled Beta distribution and *γ*_*B*_(0.5) = −0.232 for mirror lognormal. The RMSE further demonstrated that the Beta performed better than the mirror lognormal, with RMSE close to zero (7.96 × 10^−4^) for the Beta compared to lognormal (RMSE = 10.198).

**Fig 4:**
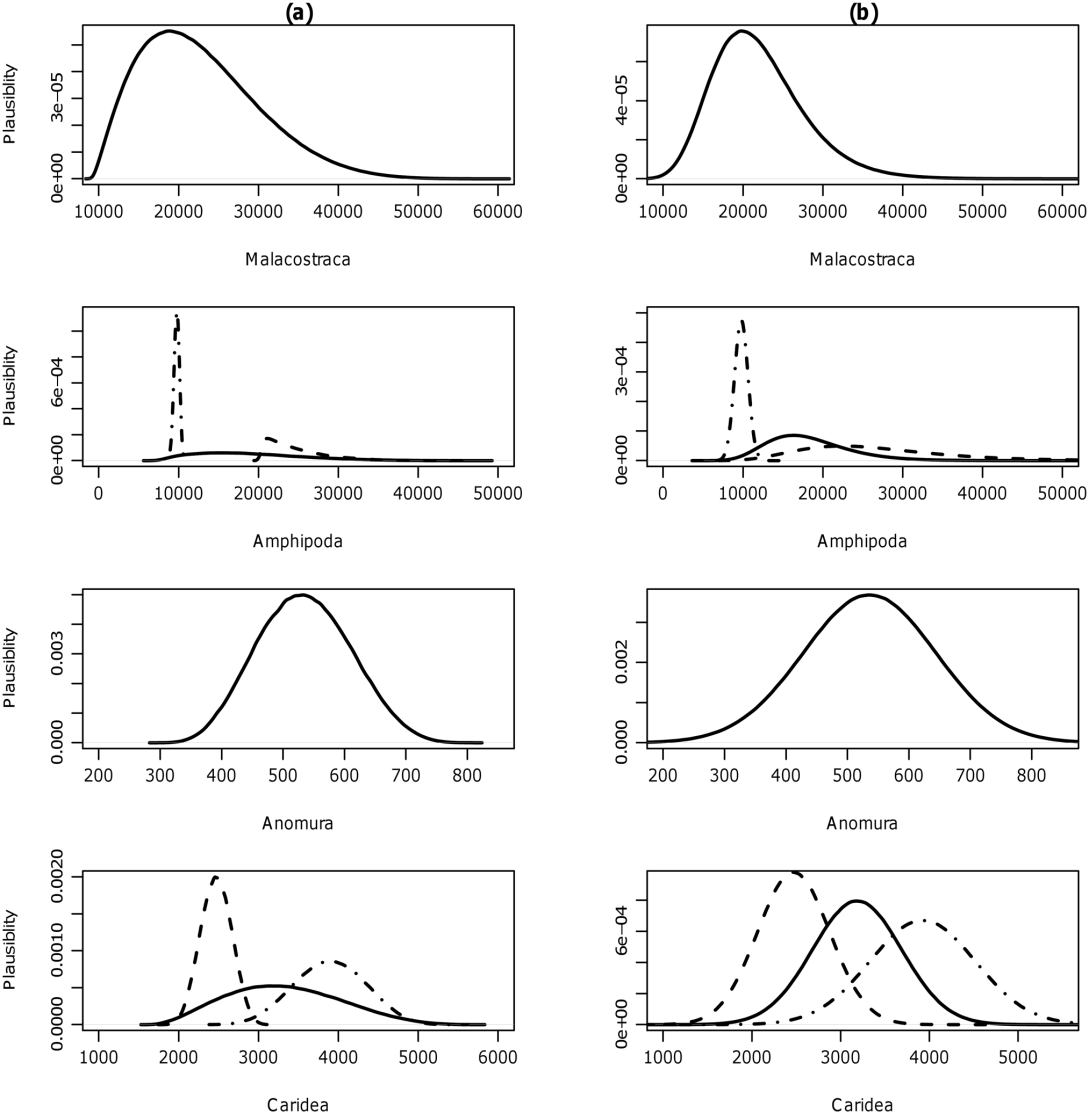
The estimated total number of species for Malacostraca and the sub-taxa. All components of all experts were encoded using (a) scaled Beta distribution and (b) Normal/lognormal distributions. The solid line is the total number of species (if there were multiple experts, then this line represents the total number of species over both experts). The dash and the dash dot lines represent the total number of species for expert one and two, respectively.

Fig 4 shows the estimated total number of species for Malacostraca and the sub-taxa, in which all components estimated by all experts were encoded using the scaled Beta distribution (Fig 4a) and Normal/lognormal distributions (Fig 4b). In the few cases where there were multiple experts, the total number of species over both experts and from each expert are also shown.

The number of species for Amphipoda assessed by the first expert was approximately double that provided by the second expert. The first expert provided numbers that were positively skewed for all components, with all but one being extremely positively skewed. The scaled Beta distribution was more positively skewed, with a skewness value of *γ*_*B*_(0.5) = 0.527 compared with *γ*_*B*_(0.5) = 0.323 for the Normal/lognormal distributions. For the second expert, three of the components were symmetric, one was negatively skewed and the other was positively skewed. Both distributions had a skewness value very close to zero. Thus the distribution for the total number of species for Amphipoda was positively skewed. The skewness value was very similar between the two distributions, with scaled Beta distribution *γ*_*B*_(0.5) = 0.283 and Normal/lognormal distributions *γ*_*B*_(0.5) = 0.264. Therefore, the total number of species for Amphipoda, over both experts, irrespective of the distribution used, encompasses both of these estimates. For Caridea, there was an overlap in the number of species between the two experts. For both experts, the uncertainty was symmetric for all components. The skewness value was very close to zero for both distributions for each expert and both experts combined. Similar to Amphipoda, the total number of species for Caridea incorporates both experts’ estimates, regardless of the distribution used.

When the expert data for a particular component reflected symmetry in their uncertainty, then the encoded mode was very similar between the two distributions. However, much larger differences were evident when the expert data was positively or negatively skewed. This difference was greater for extremely positively skewed data. For example, the first expert of Amphipoda provided a best guess of 18,000 for the undiscovered species component. The encoded mode for this component was 18,000 based on the Beta distribution (since this algorithm equates the best estimate to the mode), compared to an encoded value of 19,474 for a lognormal.

The shape of uncertainty in the total number of species for Malacostraca is similar between the scaled Beta and Normal/lognormal distributions. Both distributions resulted in a positive skewed shape, with the skewness value *γ*_*B*_(0.5) = 0.275 and *γ*_*B*_(0.5) = 0.245 respectively. For the Beta, the lower 95% credible bound, mode and upper 95% credible bound were 11,564, 18,877 and 39,384, respectively. The analogous values for the Normal/lognormal distributions were 12,882, 19,925 and 35,134. Therefore using the Normal/lognormal distributions resulted in a slightly inflated total number of species, with tighter confidence.

### Comparative study

First, we investigate how well the Normal/lognormal distributions and the scaled Beta distribution fitted the simulated expert assessments, particularly for extreme positive and negative skewness. Second, we examine the impact of various levels of sureness on the fitted and elicited values.

**Fig 5:**
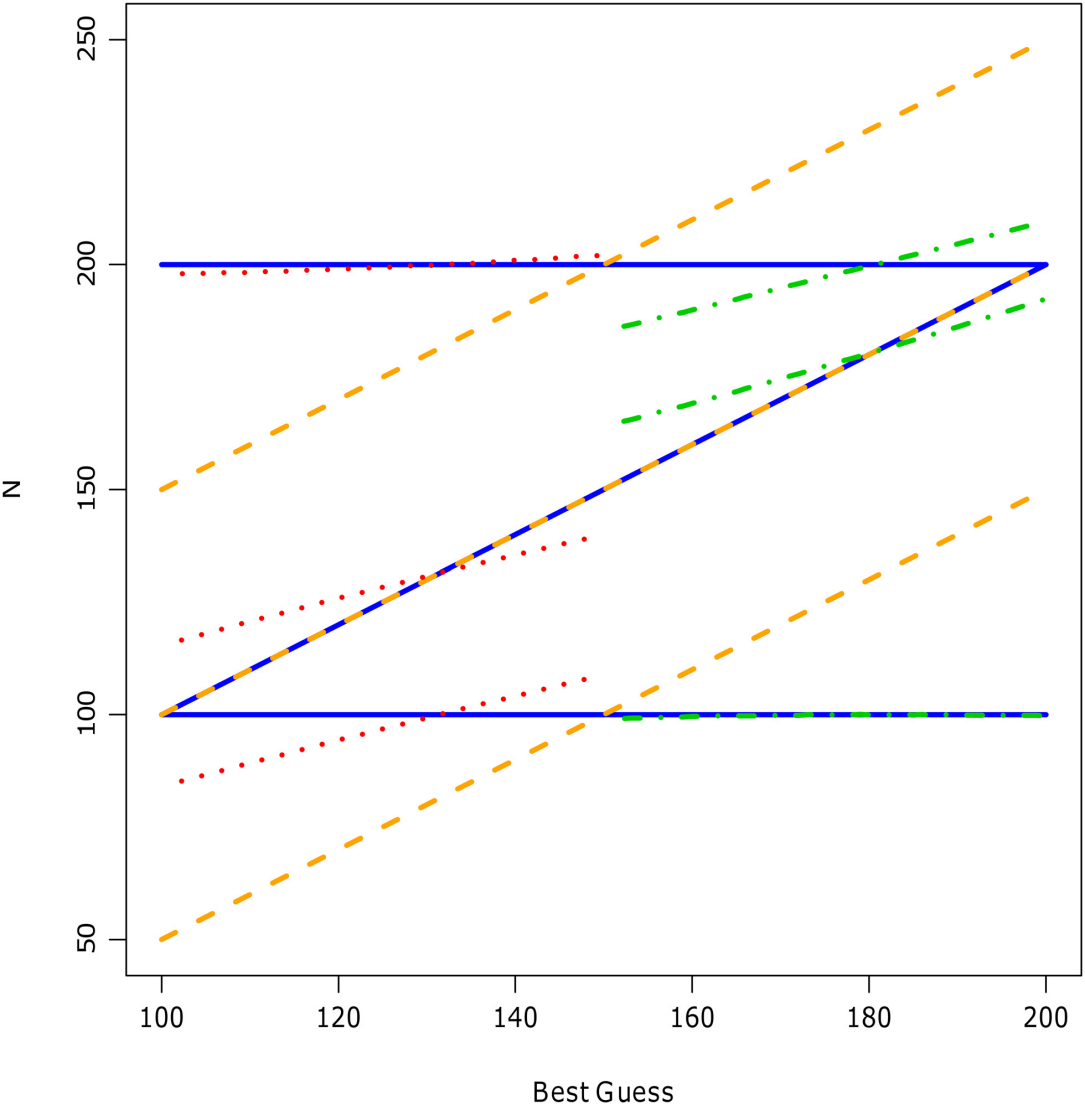
Simulated expert assessments fitted to all four distributions. Each value of the best guess (*B* a sequence between 100 and 200 incremented by one; x-axis) was fitted to scaled Beta (blue solid line), lognormal (red dot), Normal (orange dashed) and mirror lognormal (green dash dot) distributions. For each value of *B*, the simulated value of the lower (*L*) and upper (*U*) bounds are 100 and 200, respectively. The y-axis is the encoded value of *X*, i.e. total *N* number of species. For each distribution, the middle line is the encoded mode, the lower line is the encoded value of *L*, and upper line is encoded value of *U* based on 80% level of sureness (*P* = 0.80).

The results of these simulations for *P* = 0.80 are displayed in Fig 5. A similar pattern occurred for other levels of sureness. The main purpose of this figure is to show that the scaled Beta distribution accurately encodes the mode, lower and upper values (*y*-axis) for the full range of best guess values (*x*-axis). Even at the extremes when the best guess coincided with either the lower or upper values, the scaled Beta distribution encoded the three assessments accurately. In contrast, the Normal/lognormal distributions only correctly encode one of these values. By specification of the algorithm, the Normal distribution also reproduced the mode (*y*-axis) for all best guess values (*x*-axis). When the best guess was 150, then the normal distribution correctly encoded the lower and upper limits. However, as the best guess diverged from this number, i.e. as *B* moved towards the lower and upper bounds (of the 80% plausibility interval) that is 100 or 200, then the encoded values of *L* and *U* from the Normal distribution deviated from the corresponding simulated (true) assessments. For all values of *B* between the lower bound (100) and 50th percentile (150), the fitted values of *U* from the lognormal distribution more closely matched the true values than for *B* and *L*. Correspondingly, the fitted values of *L* from the mirror lognormal were very similar to the simulated lower assessment, when the best guess was between 50th percentile (150) and upper bound (200). When *B* was approximately 130, then the lognormal distribution encoded *B*, *L* and *U* accurately. Similar results were observed for the mirror lognormal when *B* was 180. When the best guess was less than 130, the encoded mode and lower numbers from the lognormal deviated substantially from the simulated expert’s numbers. Analogously, when the best guess was greater than 180, the encoded mode and upper values from the mirror lognormal also substantially differed from the simulated expert assessments.

**Fig 6:**
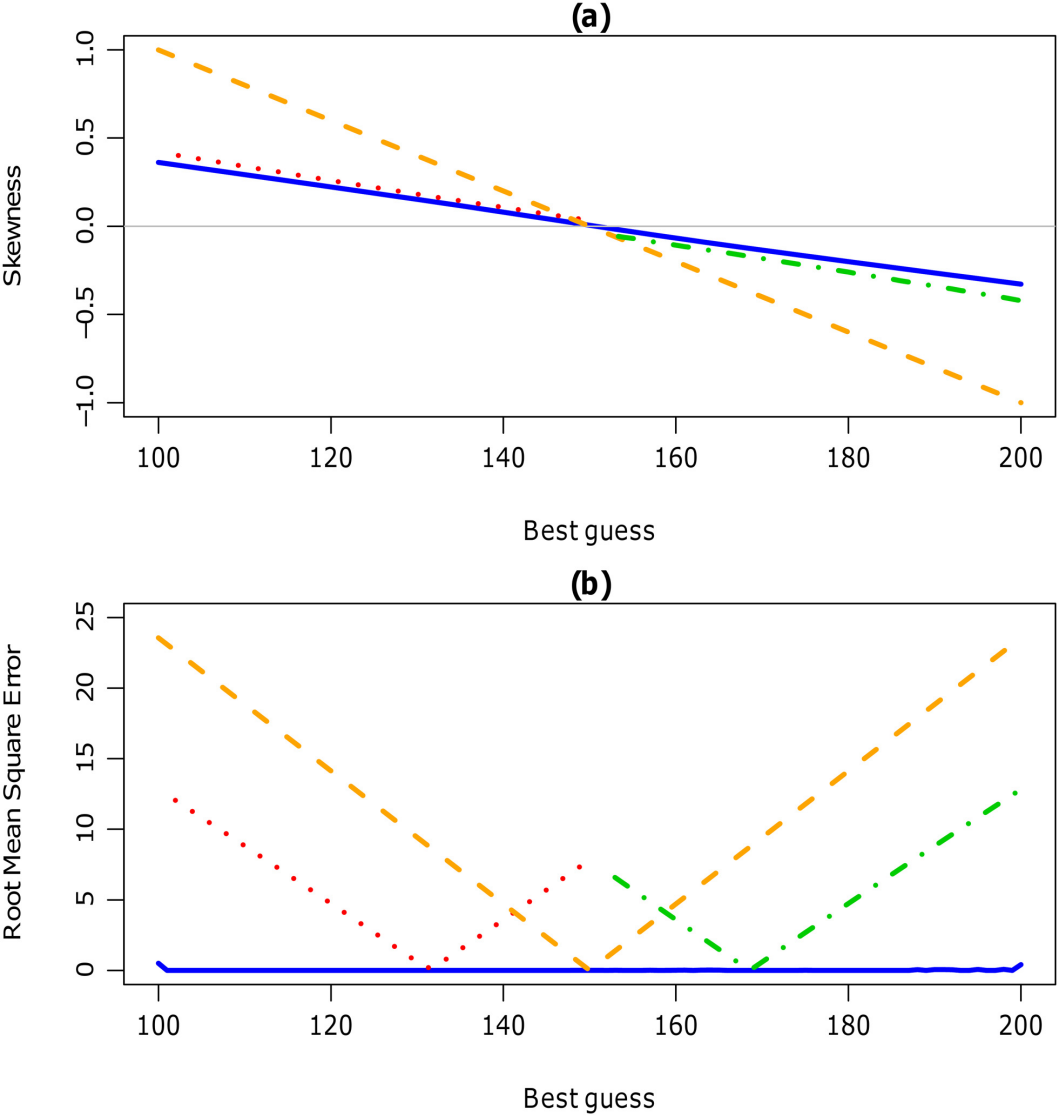
(a) Skewness and (b) RMSE for each simulated best guess for all four distributions. For each simulated best guess value between 100 and 200 (x-axis), a) skewness (*γ*_*B*_(0.5))and b) the RMSE is displayed on the y-axis from encoding the scaled Beta (blue solid) and Normal/lognormal (lognormal (red dot line), Normal (orange dashed) and mirror lognormal (green dash dot)) distributions. In a) the horizontal grey solid line denotes skewness at zero, ie. symmetric. The level of sureness *P* is 0.80, minimum is zero and maximum is 500.

For each distribution, the skewness (quantile-based measurement) was calculated for each simulated best guess, sureness, minimum and maximum. For different levels of *P*, *M*_*L*_ and *M*_*U*_, similar skewness values were obtained. Fig 6a shows the skewness for each simulated best guess for both the Normal/lognormal and scaled Beta distributions. When the best guess was equal to 150, the skewness value was very close to zero for all distributions. For all best guess values, the skewness value of the scaled Beta distribution was closer to zero, i.e. less skewed, compared to the other distributions. At extreme positive (and negative) skewness, when the best guess equalled lower (or upper) number, the Normal has a skewness of *γ*_*B*_(0.5) = 1, the lognormal has *γ*_*B*_(0.5) = 0.422, the mirror lognormal has *γ*_*B*_(0.5) = −0.422 and the Beta has *γ*_*B*_(0.5) = 0.356. Since the skewness value for the Beta was the closest to zero, thus the Beta was best at estimating the mode at extreme positive (and negative) skewness.

The RMSE was calculated for each simulated best guess, sureness, minimum and maximum, for each distribution. Similar results were obtained for different levels of *P*, *M*_*L*_ and *M*_*U*_. For both the Normal/lognormal and scaled Beta distributions, the RMSE is displayed for each simulated best guess in Fig 6b. For all best guess values, the scaled Beta distribution had a RMSE very close to zero, irrespective of the degree of skewness. The RMSE for the Beta distribution was also very close to zero for different levels of sureness, minimum and maximum values. When the simulated data exhibited extreme skewness (when *B* was equal to either *L* or *U*), then the RMSE was slightly larger, but still less than 0.5. For the Normal distribution, when the simulated values were exactly symmetric, i.e. when *B* was equal to 150, then the RMSE was close to zero but increased exponentially as *B* decreased or increased. Similarly, the RMSE values for the lognormal and mirror lognormal were almost zero when *B* was 130 and 180, respectively, but increased when *B* deviated from these values. This trend occurred for all combinations of *P*, *M*_*L*_ and *M*_*U*_.

Overall, the scaled Beta distribution performed better than the Normal/lognormal distributions, i.e. the encoded mode, lower and upper values more closely matched the values nominated by experts for different levels of sureness, different ranges between minimum and maximum, and in the presence of either positive and negative skewness. In addition, the Beta distribution was better able to cope with extreme skewness.

## Discussion

In this paper, the primary problem addressed is the estimation of the number of classes of a (hierarchical) classification. In particular, we have presented and evaluated statistical methodology for encoding of elicited information into several distributions from single and multiple experts. In our previous work [7], Normal and lognormal distributions were encoded when the expert provided answers as counts or multiplicative factors, and Beta distributions were encoded to elicited percentages. The details of encoding these distributions for elicited data were not specified in [7]. In this paper, we have provided these details and also presented an alternative distribution, the scaled Beta distribution, which can be encoded to all elicited numbers. This paper also describes how to calculate the total number of species within a group, estimate the total number across multiple experts and sum over multiple groups and experts.

The two-parameter beta has a number of appealing features with respect to representing elicited statistics as a probability distribution. It is the conjugate distribution for the Binomial distribution and is also mathematically appealing when using it as a prior for other distributions that have a probability, proportion or rate as a parameter. It is mathematically related to a range of other distributions, including the Bernoulli, triangular, arcsine, F, exponential, Dirichlet and Pearson Type 1 distributions, for specific parameter values, which again can be helpful when developing prior distributions. It is a natural descriptor for order statistics, which provides another perspective on elicited probabilities The mean, mode and variance of the two-parameter beta distribution have closed forms and, although there is no such closed form for the median, accurate approximations exist in terms of very small relative and absolute errors. The relationships between the mean, median and mode, and between the skewness and kurtosis of this distribution, are well defined. Moreover, the relative entropy, or Kullback-Leibler divergence, which measures the inefficiency of assuming incorrect parameters for the distribution, can be derived in closed form. Finally and perhaps most importantly, this distribution is very parsimonious in that has only two parameters yet can represent a very wide variety of shapes, including symmetric, U-shape, two-point, uniform, semi-elliptic, parabolic, bell-shaped, spike, skewed, J-shaped and reverse J-shaped, right triangular, straight line, and so on.

The consideration of expert judgements as deviations from an overall (two-parameter) Beta distribution is consistent with the idea of random behaviour of percentages and proportions. It is also consistent with a Bayesian perspective that each expert can indeed have a unique distribution that can be modelled by a Beta distribution with an associated uncertainty of estimation, and that these individual distributions can be combined in a formal, effectively variance-weighted, manner around an overall Beta distribution.

Given the above advantages of the Beta distribution, it is difficult to consider a more appropriate alternative for the purpose of representing the three statistics elicited from experts in our study. Exceptions are the Normal, log-normal and mirror log-normal distributions, because of their familiarity, mathematical tractability and the independence of their parameters. It is for this reason that we conducted a comparative assessment of these distributional choices.

The appeal of the scaled Beta distribution compared with the Normal and lognormal distributions is that it can capture all quantities likely to be encountered when enumerating population components, namely raw or relative counts or proportions, either in the presence of high negative or high positive skewness. Moreover, the case study and comparative studies presented in this paper indicate that this distribution performed better than the Normal and lognormal distributions over all levels of skewness. Specifically, in the case study, the encoded summary statistics from the scaled Beta distribution closely matched the actual values elicited from the expert. This distribution was also able to provide accurate encoded values under extreme skewness, for example when the experts’ best guess equalled either their lower or upper estimate. Such situations did arise during the elicitation of species richness estimates from our expert taxonomists [7]. For example, in the case of class Malacostraca, 20% of the expert’s best guesses equalled their lower estimates. In the comparative study, for all simulated best guess values, the Beta distribution was able to accurately encode the lower, mode (a best guess) and upper numbers, resulting in RMSE values close to zero. In contrast, the encoded values from the Normal and lognormal distributions were different from the simulated experts’ values and the RMSE values were very large for the majority of simulated cases. For more symmetric expert data, when the best guess was equal to or very close to the 50% quartile, accurate encoded values were obtained using all three distributions, with the RMSE values close to zero. In the case study presented here, over half (63.3%) of all data elicited were relatively symmetric.

The encoding method proposed in this paper is different to the ones provided in MATCH’s Uncertainty Elicitation Tool [27]. To clarify this we compare five features of these two approaches: A) elicitation method (in terms of the protocol, as described in [13]); B) the quantities that are elicited; C) the order in which quantities are elicited; D) the extent of skewness permitted; and E) the choice of distribution. In addressing these five features, we consider five methods for encoding a distribution provided by MATCH. Quantities elicited fall into one of five categories, leading to different forms of elicitation method (item A). These are: (1) a discrete version of the probability distribution, which is a specific version of the P-method of [13]; (2) a median and 1 or 2 quantiles with fixed probability (section 3.1.2 and 3.1.3 of [27]), which is the Q-method of [13]; (3) three probabilities for user-specified intervals (section 3.1.4 of [27]), which is the P-method of [13]; or (4) a median together with two such probabilities (section 3.1.5 of [27]), a hybrid PQ-method of [13]. These five methods thus directly correspond to the P-, Q- or PQ-methods for encoding probabilities, also reviewed in [23] and [32] and compared to the Elicitator method in [25]. In contrast, our tool asked experts to specify the mode together with two pairs of quantiles and the associated probability of each pair, which a mode-extended version of the PQ-elicitation method [13].

In terms of elicited quantities (B), our method asks the expert to specify the most plausible value, which is encoded as the mode rather than the median, and is not sought in any of the MATCH methods (1–4), but when working with ecological experts has proved more intuitive [3, 25]. Although when encoding the normal distribution, these quantities are mathematically equivalent, the wording of questions is necessarily different. For the order of elicitation (C), in contrast to MATCH and the four-point elicitation method [43], our tool enforces an “outside-in” approach to specifying the distribution [25, 26], by first asking for the lower and upper bounds, and then seeking uncertainty/sureness around these bounds, and finally asking for the mode (best guess). This helps reduce the potential to misinterpret the bounds as a confidence interval on the best estimate, rather than the desired (and hence broader) bounds on plausible range of values for the variable. In addition, regarding the extent of skewness (D), we allow for extreme skewness, where the mode is equal to the lower or upper bound. As discussed in the Introduction, there are several reasons that extreme skewness may arise in some cases, such as the motivating case study in taxonomy. For example, both the best guess and lower bound (on the number of species in a taxon) may correspond to the known published number [11]. Finally, the choices of distribution (E) both MATCH and our tool include the normal, log normal and Beta. Our tool focuses on supporting elicitation of skewed counts by also including the mirror lognormal distribution, whereas MATCH focuses on supporting elicitation of data types beyond counts and proportions, via the *t*, log *t*, and gamma distributions.

Six distributions are available in the MATCH tool for describing the elicited values, namely the normal, *t*, beta (scaled if the limits are not 0 and 1) gamma, log normal and log *t*. The software reports which distribution best fits the judgement; highly skewed judgements can lead to choice of the (scaled) Beta distribution. In the methodology underlying the MATCH tool, it was assumed that the elicited median would not equal either the elicited minimum or maximum, and hence does not allow for extreme skewness.

As detailed above the elicitation method proposed in this paper is quite different to MATCH’s Uncertainty Elicitation Tool [27], in terms of elicitation method, elicited quantities, and order of elicitation. In addition, we allow for extreme skewness, i.e. the mode being equal to the lower or upper number, which also guides the selection of different distributions for encoding.

As discussed above, this paper also details a case study focused on estimating the total number of species globally for a small subset of taxa on coral reefs. Estimation of total species richness on Earth, whether by taxon, habitat, ecosystem or the entire planet is an important and difficult problem that has defied resolution for many decades [44]. Because the taxonomy of the worldâ??s biota is still substantially incomplete [45], there is currently no opportunity to validate estimates of global species richness, rendering this a post-normal science situation as described earlier. As a consequence, global estimates of species richness must rely on some form and/or combination of functional models, species discovery curves, or the use of expert knowledge of how many species have been described and named and how many more are likely to remain to be discovered. Functional models and the use of species discovery curves, however, have proved ineffective to date if convergence on agreed estimates is used as the criterion of success [44]. In our opinion, elicitation and use of expert knowledge provides the best option currently available for achieving convergence in these estimates; see [42] for further discussion. Given the possibility that many millions of species remain to be discovered, described and named and that the pool of taxonomists available to accomplish this task is limited, harnessing expert knowledge to provide better estimates of species richness presents its own challenges.

Some of these challenges of describing expert opinion were clearly evident in the case study we presented. The distributions for the various taxa varied from strongly negatively skewed, through to symmetrical and strongly positively skewed dependent largely on the opinion of the experts about the completeness of the taxonomy of those particular groups; e.g., the more complete the taxonomy or where a high degrees of synonymy was suspected the more likely was *B* to approximate *U*. Despite differences in *B* between taxonomists elicited for a particular taxon, multiple taxonomists consistently ranked Caridea as being less than half as rich as Amphipoda. It was also evident that of the distributions fit to these data, the scaled Beta distribution proved best on a series of criteria and is therefore recommended for future applications. In addition, when multiple experts are available to provide estimates for one class or taxon they may provide, as was the case here, quite different estimates and skewnesses. These separate estimates and skewnesses must be taken into account in assembling a composite estimate for that taxon, and these taxon-specific estimates must then be combined if an estimate higher up the taxonomic hierarchy is desired. The method described here is “democratic”, in that it captures and formally combines the estimates elicited from all of the experts. Using the scaled Beta distribution makes combining estimates from multiple experts simpler.

This approach to describing expert opinion can be easily modified to suit various research questions, where the problem involves estimating (hierarchical) counts. For example, it is applicable to estimating the number of species in other terrestrial and marine environments or estimating population sizes of rare species. It is also applicable in quite different contexts, such as estimating the number of homeless people in a country. This is a well known difficult problem [14, 46–48] for which expert opinion could be a valuable complement, if it is elicited and represented accurately and adequately. We suggest that the methodology proposed here could achieve this.

## Supporting Information

S1 Text We provide additional supporting details of the MSS for lognormal and Beta distributions. (PDF)

S1 Table This table contains the Malacostraca data. First column is the expert number. Next 12 columns relate to the taxonomic classification. Last six columns contain the expert data, which includes minimum *M*_*L*_, lower *L*, best estimate *B*, upper *U*, maximum *M*_*U*_ and sureness *P*.
(XLSX)
