# Supplementary material for: Characterising Uncertainty in Expert Assessments: Encoding Heavily Skewed Judgements
Source: PLoS One. 2015 Oct 30;10(10):e0141697. doi: 10.1371/journal.pone.0141697 (PMC4627781; doi:10.1371/journal.pone.0141697)
Supplement: S1 Text — (PDF) [file pone.0141697.s001.pdf]

Log-normal For the log-normal distribution $LN(\mu, \sigma^2)$, the mean μ is $e^{\mu+\sigma^2/2}$ and the mode is $e^{\mu-\sigma^2}$ so the values of μ and σ^2 are obtained by minimizing the MSS:

$$\begin{aligned} MSS_{\text{LN}} = &((\log(L_{expert}) - \log(L_{LN_i}))^2 * \frac{1}{3}) \\ &+((\log(U_{expert}) - \log(U_{LN_i}))^2 * \frac{1}{3}) \\ &+((\log(B_{expert}) - \log(exp(\mu_i - \sigma_i^2)))^2 * \frac{1}{3}), \end{aligned} \tag{1}$$

where $i = 1, \ldots, I$ are all possible combinations of means μ_i and standard deviations σ_i tested. An analogous approach is used for the mirror log-normal distribution, when the expert data is negatively skewed.

Beta Distribution In Fisher *et al.* [1] software, if the expert answered as a percentage, then a Beta distribution was fitted. For the Beta distribution $Be(\alpha, \beta)$, the mean is $\alpha/(\alpha + \beta)$ and the mode is $(\alpha - 1)/(\alpha + \beta - 2)$, so α and β are obtained by minimizing the MSS:

$$\begin{aligned} MSS_{\text{Beta}} = &((\text{logit}(L_{\text{expert}}) - \text{logit}(L_{\text{Beta}_i}))^2 * \frac{1}{3}) \\ &+((\text{logit}(U_{\text{expert}}) - \text{logit}(U_{\text{Beta}_i}))^2 * \frac{1}{3}) \\ &+((\text{logit}(B_{\text{expert}}) - \text{logit}((\alpha_i - 1)/(\alpha_i + \beta_i - 2))^2) * 1/3), \end{aligned} \tag{2}$$

where $i = 1, \ldots, I$ are all possible combinations of α_i and β_i.

References

1. Fisher R, O'Leary RA, Low-Choy S, Mengersen K, Caley MJ. A software tool for elicitation of expert knowledge about species richness or similar counts. Environmental modelling and software. 2012;30:1–14.
